# Statistical analysis of socioeconomic and demographic correlates of perinatal mortality in Tigray region, Ethiopia: a cross sectional study

**DOI:** 10.1186/s12889-019-7642-z

**Published:** 2019-10-16

**Authors:** Berhanu Teshome Woldeamanuel, Kumachew Kusse Gelebo

**Affiliations:** 1 Department of Statistics, College of Natural and Computational Sciences, Salale University, Fiche, Ethiopia; 2 Department of Statistics, College of Natural and Computational Sciences, Mekelle University, Mekelle, Ethiopia

**Keywords:** Perinatal mortality, Early neonatal death, Stillbirth, Tigray, Ethiopia, Logistic regression

## Abstract

**Background:**

Though Ethiopia achieved the fourth Millennium Development Goal, commit to reducing under five child mortalities by the year 2015, but perinatal mortality has remained a major public health problem in Ethiopia, and the Tigray region is experiencing a high perinatal mortality rate. This study aimed to assess the risk factors attributed to perinatal death in the Tigray region.

**Methods:**

A retrospective cross-sectional study was used. The information collected from 2738 children born five years preceding the survey was considered. Variables such as maternal social and demographic characteristics, child demographic characteristics, health and environmental factors were considered as risk factors of perinatal death. The study used descriptive statistics, and logistic regression model to identify significant correlates of perinatal mortality.

**Results:**

The data showed that from total children included in the study, 4.1% are early neonatal deaths, and 2.1% are stillbirth. Overall the prevalence of experiencing perinatal mortality was 6.2% the Tigray region. The logistic analysis revealed, factors small birth interval (less than 15 months) (AOR = 7.902; 95% CI: (4.526–13.795)) and 16–26 months (AOR =2.088; 95% CI: (1.292–3.375)), poor wealth index (AOR = 1.948; 95% CI: (1.011–3.754)), having no toilet facility (AOR =1.649; 95% CI: (1.093–2.488)), child sex (being male) (AOR =1.74; 95% CI: (1.234–2.454)), giving birth at older maternal age (45–49 years) (AOR = 0.293; 95% CI: (0.128–0.668)), rural residence and using the unprotected well water were significantly associated with a higher risk of perinatal death.

**Conclusions:**

The study identified sex of a child, previous birth intervals, availability of toilet facilities, wealth index, birth type, mother’s age, parity, place of residence, mother’s occupation and source of drinking water were the factors significantly associated with perinatal mortality. The prevalence of perinatal mortality shows that Tigray region was experiencing a high perinatal mortality rate than the national.

## Background

World health organization (WHO) defines perinatal mortality as neonatal deaths within the first week of life and fetal deaths after 28 weeks of gestation. The perinatal mortality rate (PMR) is the number of perinatal deaths per 1000 total births. It is an indicator of the social, economic and environmental conditions in which mother lives, including maternal health care utilization. It is multi factorial in etiology indicators of health status of the community and mainly depends on the quality of health care services mothers get during pregnancy and the new born babies [1].

Out of about 6 million perinatal deaths occur globally, 3.3 million were stillbirths and the majority (more than 97%) occurs in low and middle income countries, in South Asia and sub-Saharan countries [2]. In developing countries, the survival rate of preterm newborns is very low. Perinatal mortality rate (PMR) is five times higher (50 per 1000 total births) in developing countries than that of in developed countries (10 per 1000 total births) [3].

Perinatal mortality is still a major public health issue. Underlying major cause of deaths of children is essential to achieve significant improvement in public health and in controlling maternal and child mortality. Thus, to improve the health status of mothers’ and newborn policymakers has set guidelines and address these risk factors with proper intervention. Framing proper guidelines and policies to reduce child mortality will insure the sustainability in achieving the reduction in early neonatal mortality [3].

The more risk factors of perinatal death and stillbirth are preterm birth, low birth weight, fetal growth restriction and congenital abnormalities [4]. Thus, to reduce the high mortality rate of a child and incidence of complications at the early neonatal period and to ensure the survival of newborn infants, high-quality antenatal care, professional delivery assistance and postnatal care should be taken.

In Ethiopia Infant and under-five mortality rates evidence a continuous declining trend in the past fifteen years. On the other hand, even though perinatal and neonatal mortality rate decreased from 46 deaths per 1000 pregnancies in 2011 to 33 deaths per 1000 pregnancies in 2016 and 37 deaths per 1000 live births in 2011 to 29 live births in 2016, it has remained stable high. According to the 2016 Ethiopia Demographic and Health Surveys (EDHS), the Tigray region is experiencing a higher perinatal mortality rate of 36 per 1000 pregnancies, compared to that of the national average rate of 33 per 1000 pregnancies [5, 6].

Ethiopia is one of the countries achieved the fourth Millennium Development Goal (MDG4) commit to reducing under five child mortalities by the year 2015. Even there is a high decrease in child mortality; perinatal mortality remains high in the country. Thus, a considerable reduction in perinatal and neonatal deaths is crucial to achieve this goal more in the future. Previous research works stated that decline in early neonatal mortality over the last three decades has been slower than declines in late neonatal (deaths occurring after the first week of life, but before the first month of life) mortality.

Interventions to reduce perinatal mortality are an important concern and belong to improving maternal health care, where it is essential for monitoring the current health programs and formulating policies on improving the current situation for maternal and child health. Additionally, knowledge of the factors associated with perinatal mortality is, therefore, essential to monitor the development of intensive and evidence-based health interventions to avoid neonatal deaths [7, 8].

Despite the fact that a number of researches have been done on the identification of factors that are associated with infants and neonatal mortality in Ethiopia, there are limited studies that look at the PM and its determinants. The majority of them are concentrated on the national level. Such studies miss an important point of policymakers as the national result may not show the exact situation at regional levels. To address this gap, we conducted an all-inclusive cross-sectional analysis of the recent 2016 Ethiopian Demographic Health Survey, to explore the major risk factors of perinatal mortality in the Tigray region, taking into consideration various demographic, socioeconomic and environmental factors in the Tigray region, Ethiopia [5]. Therefore, the main aim of this study was to assess the determinant factors of perinatal deaths in Tigray region, Ethiopia.

The significance of this study is the research outcome will be helpful to strength national policies and intervention strategies targeted at reducing early neonatal death and stillbirth in Ethiopia. The research will also serve as a baseline for other researchers who are going to undertake further study in the area.

## Methods

### Source of data and description of the study area

This study was based on secondary analysis of retrospective cross-sectional study based on data from 2016 Ethiopian Demographic and Health Survey. The survey was carried out by the Central Statistics Agency (CSA), the Ministry of Health (MOH) and the Ethiopian Public Health Institute from January 18, 2016 - June 27, 2016. It was funded by the United States Agency for International Development (USAID). The data were obtained from the DHS measure Program with permission [9]. The main aim of this survey is to deliver detailed information on fertility, family planning, infant, child, adult and maternal mortality, maternal and child health, nutrition and knowledge of HIV/AIDS and other sexually transmitted infections to policymakers and planners.

The survey had information on a range of socioeconomic and demographic factors of the population nationwide. It implemented a two-stage sample design. The country has nine regions and two administrative cities. In the first stage 645 enumeration areas (202 in urban areas and 443 in rural areas) were selected with probability proportional to size. Second, stage involved selection of 28 households per cluster of equal probability systematic selection of the newly formed household list. The EDHS 2016 has three questionnaires: the household questionnaire, the woman’s questionnaire and the man’s questionnaire. All women of childbearing age (15–49 years) and who were either stable residents of the chosen households or visitors, who lived in the household at least one night before the survey, were eligible for the interview. Data were collected by face-to-face interviewing women that met the eligibility criteria.

A total of 15,683 women of the reproductive age group, 12,688 men aged 15–59 years in 16,650 households were interviewed in 2016 EDHS from these, 1682 households were from Tigray region. In the region 4428 births were reported in the last five years preceding the survey. Next, child with information on early neonatal death and stillbirth during the last five years were identified [10]. Finally, a total of 2738 children that have completed information about all the factors considered were included.

Tigray region is located between 36 and 40 degrees east longitude north of Ethiopia bounded in the north with Eritrea, in the east Afar region, the south Amhara region and the Sudan in the west. The 2007 Ethiopian population and housing censuses reported that the Tigray region had a population size of 3,136,267 of which 1,594,102 was females. About 85% (2,667,789) of the population are rural residents [11].

### Variables of the study

Determinants of perinatal deaths in this study were selected from the available prior studies on the subject. Important mother and child demographic characteristics explored from a theoretical perspective and various literatures for perinatal survival are presented in Table 1 [12–15]. Wealth index was coded as: 1 = poor (includes poorest and poor), 2 = middle and 3 = rich (include richer and richest). The two categories have been merged since the percentage distribution of events (perinatal death) in the poorest and richest categories is too small. Similarly, the percentage of women in the age category 15–19 and 20–24 after screening for missing variables is too small and authors merge these two categories with age group of 25–29 years old. Since this study included only mothers who gave birth in the past five years preceding the survey, the percentage of mothers who gave birth between ages 15–24 are too small. This too few frequencies may, in turn, affect the parameter estimation. Table 1.

**Table 1:** Operational definition and categorization of independent variables used in the study

S.No	Variable	Description and codding
1	Sex	Sex of perinatal: 0 = male, 1 = female
2	Type of birth	Type of perinatal birth, 0 = single, 1 = multiple
3	Maternal age	Mother’s age at child birth: 1 = 29 and below, 2 = 30–34, 3 = 35–39, 4 = 40–44, 5 = 45–49
4	Residence	Place of residence: 0 = urban, 1 = rural
5	Maternal education	Mother’s education level: 1 = no education, 2 = primary, 3 = secondary and higher
6	Previous pregnancy interval	Previous pregnancy interval in months: 1 = 15 and below, 2 = 16–26, 3 = 27–38, 4 = 39 and above
7	Wealth index	Family health index: 1 = poor, 2 = middle, 3 = rich
8	Mother job	Mother’s occupation 1 = Not working 2 = non agriculture sector 3 = agricultural sector
9	HIV status	Maternal HIV status: 0 = no, 1 = yes
10	Birth order	Birth order of child: 1 = 3 and below, 2 = 2–5, 3 = 6 and above
11	Parity	Total children ever born:1 = 3 and below, 2 = 4–5, 3 = 6–8, 4 = 9 and above
12	Source of drinking water	Source of drinking water: 1 = piped water, 2 = protected well, 3 = unprotected well
13	Toilet facilities	Toilet facilities of mothers: 0 = No facility/bush/field, 1 = with facilities
14	Sex of household head	Sex of the household head: 0 = male, 1 = female
15	Paternal education	Husband’s education level: 1 = no education, 2 = Primary, 3 = secondary and higher

### Dependent variable

During the conduct of the DHS, mothers were asked to report any pregnancy lost and death of a live born infant. Then, the perinatal mortality will be obtained as the sum of the death of infants within the first week of life and pregnancy loss occurring after seven completed months of gestation. The dependent variable of this study was perinatal death (stillbirth and early neonatal mortality). Thus, the outcome variable for the i^th^ child is dichotomous, represented by a random variable Y_i_ that takes the value “1” with probability of success (perinatal death) and the value “0” with probability of failure (no perinatal death), such that

$$ {Y}_i=\left\{\begin{array}{c}1\  if\ there\ is\ perinatal\ death\ \\ {}0\  if\ there\ is\  no\  perinatal\ death\end{array}\right. $$

### Statistical methods of data analysis

The study used descriptive statistics such as frequency distributions and percentages to describe the sample information. Multivariate logistic regression was used to estimate the effect of socioeconomic and demographic factors on perinatal death (odds ratios with their 95% confidence intervals). The goodness of fit test was done using the likelihood ratio test (LRT) and Hosmer_lemeshow tests. The degree of collinearity was checked using the variance inflation factor (VIF).

## Results

### Descriptive statistics

To identify the risk factors associated with perinatal mortality, 2738 births within the five years before the survey was considered. The result from Table 2 showed that out of 2738 births included in the study, 170 (6.2%) were reported perinatal deaths, of which 111 (4.1%) were early neonatal deaths and 2.1% were the stillbirths. This shows that the ratio of early neonatal deaths to stillbirths is 2/3 to 1/3. The possible explanation for this variation might be that stillbirths are likely being missed as women are afraid of reporting pregnancy terminations (fetal deaths) due to some cultures and traditions in the community. Similarly, from the total population of perinatal deaths 65.3% were early neonatal deaths (deaths before one week of a life after birth) and 34.7% were stillbirths (fetal death after 28 weeks of gestational periods). Therefore, the analysis finds that the prevalence of perinatal mortality rates was 62 per 1000 births for the five years preceding the survey.

**Table 2:** Distribution of perinatal mortality by selected maternal socioeconomic, demographic characteristics, child demographic characteristics, and health and environmental factors in Tigray (*n* = 2738)

Covariates	Categories	Distribution of children		Perinatal death	
		N	%	N	%
Maternal age	29 and less	366	13.4	24	6.6
	30–34	493	18	21	4.2
	35–39	756	27.6	38	5
	40–44	623	22.8	36	5.8
	45–49	500	18.3	51	10.2
Maternal education	No education	2074	75.7	124	6
	Primary educ.	555	20.3	38	6.8
	Secondary/higher	109	4.0	8	7
Husband education	No education	1537	56.1	102	6.6
	Primary educ.	1012	37	55	5.4
	Secondary/higher	189	6.9	13	6.9
Sex of household head	Male	2573	94	149	5.8
	Female	165	6	21	12.7
Sex of child	Male	1405	51.3	108	7.7
	Female	1333	48.7	62	4.6
Residence	Urban	309	11.3	11	3.6
	Rural	2429	88.7	159	6.5
Birth interval	15 and less	154	5.6	120	77.9
	16–26	633	23.1	50	7.9
	27–38	992	36.2	51	5
	39 and more	959	35	35	3.6
Birth order	3 and less	1266	46.2	86	6.8
	4–5	814	29.7	40	4.9
	6 and more	658	24	44	6.7
Source of drinking water	Piped water	683	24.9	33	4.8
	Protected well	1224	44.7	71	5.8
	Unprotected well	831	30.4	66	7.9
Toilet	No toilet facility	1495	54.6	116	7.8
	With toilet facility	1243	45.4	54	4.3
Wealth index	Poor	914	33.4	75	8.2
	Middle	1122	41	69	6
	Rich	702	25.6	26	3.7
Parity	3 and less	342	12.5	29	8.5
	4–5	632	23.1	25	4
	6–8	1274	46.5	60	4.7
	9 and more	490	17.9	56	11.4
Mother’s HIV status	No	2280	83.3	147	6.4
	Yes	458	16.7	23	5
Maternal occupation	Not working	992	36.2	50	5
	Agricultural sector	575	21	53	9.2
	Non-agricultural sector	1171	42.8	67	5.7
Total perinatal death				170	6.2
Early neonatal				111	4.1
Stillbirth				59	2.1

Table 2 presented the percentage distribution of the selected risk factors of perinatal mortality in the study. About one-fourth of mothers’ were in the age group 35–39 (27.6%), and nearly one-tenth of mothers’ were below or equal to 29 years, 18 and 18.3% were in other age groups 30–34 and 45–49, respectively. More than three-fourth (76%) of mothers were illiterate (have no education at all), and only one-fifth (20%) and 4% of the mothers were attained a primary and secondary or higher education. Regarding partner’s or husband’s education level, more than half (56%) were illiterate, 37 and 6.9% of husbands attained a primary and secondary or higher education, respectively. On the other hand, when the sex of household head was concerned, 94% were reported male household head.

About one-third (33.4%) of the births in the study had belonged to a woman with poor wealth index, one-fourth (25.6%) were belonged to rich wealth index and 41% were in the middle wealth index group. Concerning mother’s occupation, nearly half of the mothers were working in the non-agricultural sector, 36.2% did not work in any sector and 21% were working in the agricultural sector.

The majority of the mothers (46.5%) had 6–8 children in their lifetime, 17.9% of respondents had at least eight children and 12.5% had less than four children in their life. Approximately half of the study population had birth orders three or less (46.2%), 29.7% had 4–5 birth orders and 24% had six or more birth orders. Regarding the previous birth intervals, 5.6% had 15 or less months of birth intervals, 23.1, 36.2 and 35% had a birth interval of 16–26, 27–38 and at least 39 months, respectively. Table 2 reveals that more than three-fourth (88%) of the respondents resided in rural areas. It also indicates that nearly one-third of respondents was using the unprotected well water for drinking and more than half (54.6%) had no toilet facilities.

The highest proportion of perinatal deaths occurs in the mothers of age groups 45–49, 35–39 and 40–49, respectively. More mothers get older more they experience perinatal deaths. Mothers with no education at all experienced the highest percentage of perinatal mortality (4.5%). As far as partner educated is concerned, the proportion of perinatal death decreased with an increase in the education level of partner.

Wealth index was inversely proportional to perinatal mortality, as wealth index increases from poor to middle and then to rich, perinatal mortality decreases from 2.7 to 0.9%. Thus, those infants born to low wealth index groups have the highest percentage of perinatal mortality than any of the higher wealth index group. Children belong to a mother who work in non-agricultural sector had a relatively higher percentage (2.4%) of perinatal mortality.

The percentages of perinatal mortality were the highest among women that had at least nine children (11.4%). A child with the smallest birth order had the highest chance of being died the first week of life. Similarly, children with birth intervals less than 15 months encountered the highest percentage (77.9%) of perinatal mortality.

Mothers living in urban were educated and had a higher access to public health care services thus, they were aware of the benefit of using this health care services in keeping the health of their babies than mothers of rural areas. The percentages of perinatal deaths in rural and urban areas were about 6.5 and 3.6%, respectively. Concerning sanitation indicator variables, the proportions of perinatal mortality was higher among mothers using the unprotected drinking water than that of piped drinking water users. Similarly, perinatal mortalities were higher among mothers with no toilet facility/bush/field than that of with facilities. Table 2.

### Binary logistic regression results

Binary logistic regression analysis was used to examine the effect of each covariate on the perinatal death.

### Assessment of goodness of fit of the model

We start here first by checking the overall goodness of fit using the LRT and Hosmer -Lemeshow test. Accordingly, the likelihood ratio test, provided a chi-square value of 406.618 (*p*-value < 0.0001), which would imply good fit for the model. Similarly, the Hosmer-Lemeshow test is found the observed data was better explained by the model (chi-square value = 7.577 with 8 degrees of freedom and *p*-values = 0.476). Further, since the VIF value for all predictors is < 10 there is no collinearity problem.

### Interpretation of logistic regression results

The multivariate analysis of risk factors associated with perinatal mortality was presented in Table 3. The result showed birth type, sex of a child, previous birth intervals in months, maternal age, place of residence, mother’s education level, wealth index, source of drinking water, availability of toilet facilities, sex of household head, parity and maternal occupation were statistically significant predictors of perinatal death. Whereas birth order, husband/partner education level and maternal HIV status were not statistically significant.

**Table 3:** Factors associated with perinatal mortality: crude (unadjusted) and adjusted odds ratio estimates of logistic regression analysis

Covariates	Crude (unadjusted)				Adjusted			
	OR	95% C.I		p-value	AOR	95% C.I		p-value
Birth type								
Multiple birth	1.00				1.00			
Single birth	0.190	0.113	0.319	< 0.0001*	0.179	0.100	.321	< 0.0001*
Sex of child								
Female	1.00				1.00			
Male	0.586	0.425	0.808	0.001*	1.740	1.234	2.454	0.002*
Birth interval								
39 and more months	1.00				1.00			
15 and less month	7.480	4.497	12.442	< 0.0001*	7.902	4.526	13.795	< 0.0001*
16–26 months	2.264	1.452	3.530	< 0.0001*	2.088	1.292	3.375	0.003*
27–38 months	1.431	0.922	2.221	0.110	1.509	0.951	2.396	0.081
Birth order								
6 and more	1.00				1.00			
3 and less	1.071	0.698	1.481	0.930	1.231	0.767	1.975	.389
4–5	0.721	0.464	1.121	0.146	0.977	0.600	1.592	.927
Maternal age								
45–49 years	1.00				1.00			
29 & less year	0.618	0.373	1.024	0.062	0.293	0.128	0.668	0.003*
30–34 years	0.392	0.232	0.662	< 0.0001*	0.335	0.173	0.646	0.001*
35–39 years	0.466	0.301	0.721	0.001*	0.381	0.231	0.628	< 0.0001*
40–44 years	0.540	0.346	0.842	0.007*	0.585	0.360	0.948	0.029*
Residence								
Rural	1.00				1.00			
Urban	0.527	0.283	0.982	0.044*	0.364	0.136	0.970	0.043*
Mother Education								
Secondary and Higher	1.00				1.00			
No education	0.803	0.382	1.687	0.562	0.496	0.170	1.447	0.199
Primary education	0.928	0.420	2.048	0.853	0.861	0.302	2.457	0.780
Source of water								
Unprotected	1.00				1.00			
Piped source	0.588	0.383	0.905	0.016*	0.675	0.382	1.193	0.176
Protected well	0.714	0.504	1.010	0.057	0.684	0.469	0.997	0.048*
Toilet facility								
With toilet	1.00				1.00			
No toilet facility	0.540	0.387	0.753	< 0.0001*	1.649	1.093	2.488	0.017*
Sex of household head								
Female	1.00				1.00			
Male	0.421	0.259	0.686	0.001*	0.378	0.213	0.670	0.001*
Wealth index								
Rich	1.00				1.00			
Poor	2.324	1.471	3.672	< 0.0001*	1.948	1.011	3.754	0.046*
Middle	1.704	1.074	2.702	0.024*	1.540	0.828	2.865	0.172
Parity								
9 and more children	1.00				1.00			
3 & less	0.718	0.448	1.150	0.168	1.065	0.447	2.541	0.887
4–5	0.319	0.196	0.520	< 0.0001*	0.418	0.211	0.828	0.012*
6–8	0.383	0.262	0.560	< 0.0001*	0.401	0.257	0.626	< 0.0001*
Husband education								
Secondary/Higher	1.00				1.00			
No education	0.962	0.529	1.750	0.900	0.517	0.219	1.220	0.132
Primary education	0.778	0.416	1.454	0.432	0.533	0.230	1.235	0.142
HIV status								
Yes	1.00				1.00			
No	0.767	0.488	1.205	0.250	1.435	0.842	2.446	0.184
Mother occupation								
Agriculture	1.00				1.00			
Not working	0.875	0.600	1.274	0.485	1.053	0.693	1.599	0.809
Non-agriculture	1.673	1.150	2.434	0.007*	2.734	1.773	4.216	<.0001*

*Significant at *p* < 0.05,

Note: LRT = 406.618 (*p* < 0.001),

Hosmer and Lemeshow Test = 7.577 (*p* = 0.476)

According to the results from Table 3, the odds of perinatal mortality were lower among children that were singletons than those in multiple births (AOR = 0.179; 95% CI: (0.1–0.321)). Compared to a female child, males were more likely to die before celebrating their first week of life keeping all other variables constant. The odds of perinatal mortality were 74% times higher among male children than the females (AOR = 1.74; 95% CI: (1.234–2.454)).

Concerning birth interval, the odds of perinatal mortality were higher among children with a previous birth interval of less than or equal to 15 months and previous birth intervals of 16–26 months (AOR = 7.902; 95% CI: (4.526–13.795)) and (AOR = 2.088; 95% CI: (1.292–3.375)), respectively, compared to that of a previous birth interval at least 39 months. There was no significant difference in the risk of perinatal death between birth intervals 27–38 months and 39 months or higher birth interval. Table 3 also shown that the odds of perinatal mortalities were lower among children born to mothers’, whose age were less than or equal to 29, 30–34, 35–39, and 40–44 (AOR = 0.293; 95% CI: (0.128–0.668)), (AOR = 0.335; 95% CI: (0.173–0.646)), (AOR = 0.381 95% CI: (0.231–0.628)) and (AOR = 0.585; 95% CI: (0.360–0.948)), respectively, compared to that of 45–49 years old.

Place of residence was found significantly associated with perinatal mortality. The odds of perinatal death were about 64% times lower among children in urban areas than that of the rural residents (AOR = 0.364; 95% CI: (0.136–0.970)). Availability of toilet facility had a negative impact on perinatal death. The odds of perinatal mortality were 65% times higher among children belonged to a mother without toilet facilities than that of those with toilet facilities (AOR = 1.649; 95% CI: (1.093–2.488)).

For the variable sex of household head, the odds of perinatal mortality were about 62% times lower to children from the male household head than the female counterpart (AOR = 0.378; 95% CI: (0.213–0.670)). As far as parity is concerned, the odds of perinatal mortality were 85% times lower among children, whose mothers had 4–5 total children ever born and 60% time lower among children whose mothers had 6–8 total children ever born compared to that of whose mothers who had at least nine children ever born (AOR = 0.148; 95% CI: (0.211–0.828)) and (AOR = 0.401; 95% CI: (0.257–0.626)) respectively.

Finally, occupation of the mother was found a significant predictor of perinatal mortality. Compared to children whose mothers were working in the agricultural sector, those children whose mothers were working in non-agricultural sectors had significantly higher odds of perinatal mortality (AOR = 2.734; 95% CI: (1.773–4.216)). Table 3

## Discussion

The study empirically investigated and identified the factors that were associated with the risk of perinatal mortality in the Tigray region, using the Ethiopian demographic and health survey data.

The odds of perinatal mortality were less likely among singletons than multiple births. Compared to female child, male, children were more likely to experience perinatal death keeping other variables constant. Similar previous studies done in Umguza and Bubi rural areas also showed significant association between perinatal mortality and sex of a child [16].

Birth intervals also significantly associated with perinatal death. The odds of perinatal mortality were higher for a child with short previous birth intervals. This implies that perinatal mortality decreases significantly with increasing in the previous birth interval. On the other hand, there were no significant differences in the risk of perinatal death between birth intervals of 27–38 months and 39 months or higher birth interval. This finding is consistent with previous studies [12, 16] which show a significant association with birth intervals and perinatal death. Table 3 also shows that children born to the mothers with older age had a significantly higher risk of perinatal mortality. This further indicates that women of older ages were vulnerable to experiencing early neonatal death. Previous research had also reported a significant association between perinatal mortality and maternal age [16].

Place of residence was found significantly associated with perinatal mortality, such that children from rural areas had a higher risk of perinatal death compared to that of urban areas. Prior, studies also reported that urban residents had better wealth, better access to sanitation services, better health care services and media access for health service utilization for their newborn [15].

For the variable sex of household head, the odds of perinatal mortality were lower among male household heads than that of female household heads. The odds of perinatal mortality were the lowest among children from mothers who had less number of total children ever born [16, 17].

Wealth index was another important socioeconomic variable that affects perinatal mortality in Tigray. Children born from poor wealth index group were founded, at a higher risk of being dying before celebrating their first week births than that of children from rich wealth index. Previous literature indicated that better of households had better access to higher cash incomes than that of poor wealth, allowing them a quality diet, better access to medical care [15, 16].

Another important demographic factor significantly associated with perinatal mortality in was sex. Male children were at a higher risk of perinatal death. A similar studies in the literature argued that this could be due to genetic differences between male and female children [16].

Source of drinking water, and toilet facilities were also important factors significantly associated with perinatal death in the Tigray region. Children, whose families were using unprotected drinking water, were more likely to die in the early neonatal period than that of those who use pipe water. This is because of access to unsafe water is regarded as the main cause of infectious diseases such as diarrhea and intestinal parasites [17]. Further, availability of toilet facility had a negative impact on perinatal death. Occupation of mother was also found a significant covariate of perinatal mortality. Compared to children whose mothers were working in the agricultural sector those children whose mothers were working in the non-agricultural sectors had a significantly higher risk of perinatal mortality [18, 19].

Generally, the trend showed that even though Ethiopia has achieved the MDG, in perinatal mortality, but the progress is very slow in the Tigray region.

### Strength and limitations of the study

This study used a nationally representative survey dataset, which enhance inferences for the entire country level. However, this study is based on secondary data the major limitation is; the study was a mother’s recall of events that took place for the past five years preceding the survey, which is subject to recall bias. Probably this may be women are afraid of reporting pregnancy terminations (fetal deaths) due to some cultures and traditions in the community.

## Conclusions

This study was conducted to assess correlates of perinatal mortality in the Tigray regional state. Both descriptive statistics and binary logistic regression were used to analyze the data. The Tigray region has experienced a higher perinatal mortality rate than that of the national level. The logistic regression analysis revealed that being male child, mothers working in non-agricultural sectors, previous birth intervals less than 15 months and 16–26 months, and the poor wealth index were found as factors that positively related to the risk of perinatal mortality. On the other hand, single births, mother’s age less than 45, a male household head, being urban residence and using the protected well drinking water were found factors that inversely correlated with perinatal mortality. However, variables birth order, mother’s education, husband/partner education, and maternal HIV status were found insignificant.

Hence, health care providers should give special attention and immediate care to mothers giving multiple newborn during delivery. Additionally, they should have to give awareness about birth intervals to eliminate deaths due to a short birth interval, and community based longitudinal study would be helpful to get other unmeasured risk factors.

## Data Availability

The datasets used and/or analysed during the current study are available from the corresponding author on reasonable request.

## Abbreviations

AIDS: *Acquired immune deficiency syndrome*
*CI*: *Confidence Interval*
CSA: Central Statistical Agency
DHS: Demographic and Health Survey
EDHS: Ethiopian Demographic and Health Survey
HIV: *Human Immune Virus*
MDG: Millennium Development Goal
MOH: Ministry of Health
*OR*: *Adjusted Odds ratio*
*OR*: *Odds ratio*
PM: Perinatal Mortality
PMR: Perinatal Mortality Rate
USAID: United States Agency for International Development
VIF: Variance Inflation Factor
WHO: World Health Organization

## References

[1] Lawn J, Shibuya K, Stein C (2005). No cry at birth: global estimates of intrapartum stillbirths and intrapartum-related neonatal deaths. Bull World Health Organ.

[2] Neonatal mortality, risk factors and causes: A prospective population-based cohort study in urban Pakistan. Jehan I, Harris H, Salat S, Zeb a, Mobeen N, pasha O, McClure EM, Moore J, Wright L, Goldenberg RL bull world health organ. 2009 Feb; 87(2):130–138. 10.2471/BLT.08.050963 PMC2636189 19274365

[3] World Health Organization (2006). Neonatal and perinatal mortality: country, regional and global estimates.

[4] Allanson, E. R., Muller, M., & Pattinson, R. C. (2015). Causes of perinatal mortality and associated maternal complications in a south African province: challenges in predicting poor outcomes. BMC pregnancy and childbirth, 15, 37. 10.1186/s12884-015-0472-94. PMC4339432 25880128

[5] Central Statistical Agency [Ethiopia] and ICF International (2012). Ethiopia demographic and health survey 2011.

[6] Oestergaard MZ, Inoue M, Yoshida S, Mahanani WR, Gore FM, et al. Neonatal mortality levels for 193 countries in 2009 with trends since 1990*: A systematic analysis of progress, projections, and priorities*. PLoS Med. 2011;8(8). 10.1371/journal. pmed.1001080. 10.1371/journal.pmed.1001080 PMC3168874 21918640

[7] Black RE, Cousens S, Johnson HL, Lawn JE, Rudan I, Bassani DG, Jha P, Campbell H, Walker CF, Cibulskis R, Eisele T, Liu L (2010). Mathers C; (2010): child health epidemiology reference group of WHO and UNICEF. Lancet..

[8] Lawn JE, Lee ACC, Kinney M, Sibley L, Carlo WA, Paul VK, Pattinson R, Darmstadt GL (2009). Two million intrapartum-related stillbirths and neonatal deaths: where, why, and what can be done?. Int J Gynaecol Obstet.

[9] Measure DHS, The DHS. Program: demographic and health surveys. Rockville: ICF Macro. Available at https://dhsprogram.com/data/availabledatasets.cfm.

[10] Central Statistical Agency (CSA) [Ethiopia] and ICF. 2016. *Ethiopia Demographic and Health Survey 2016*. Addis Ababa, Ethiopia, and Rockville, Maryland, USA: CSA and ICF.

[11] Central Statistical Agency, 2007 *Census* of *Population.*

[12] Yemisrach Getiye and Mesganaw Fantahun. Factors associated with perinatal mortality among public health deliveries in Addis Ababa, Ethiopia, an unmatched case control study. BMC Pregnancy and Childbirth (2017) 17:245 DOI 10.1186/s12884-017-1420-7 10.1186/s12884-017-1420-7 PMC5530490 28747161

[13] Pramesh Raj Ghimire, Kingsley E. Agho, Andre M. N. Renzaho, Monjura K. Nisha, Michael Dibley and Camille Raynes-Greenow. Factors associated with perinatal mortality in Nepal: evidence from Nepal demographic and health survey 2001–2016. BMC Pregnancy and Childbirth (2019) 19:88 10.1186/s12884-019-2234-6 10.1186/s12884-019-2234-6 PMC6417106 30866847

[14] Gulam Muhammed Al Kibria, Vanessa Burrowes, Allysha Choudhury, Atia Sharmeen, Swagata Ghosh, Arif Mahmud and Angela KC. Determinants of early neonatal mortality in Afghanistan: an analysis of the Demographic and Health Survey 2015. Globalization and Health (2018) 14:47 10.1186/s12992-018-0363-8 10.1186/s12992-018-0363-8 PMC5944060 29743085

[15] Oji OS. Risk factors for perinatal mortality in Nigeria: the role of place of delivery and delivery assistant. JULY. 2011;2008.

[16] Mbiba V (2015). Factors associated with perinatal mortality in Umguza and Bubi rural areas, 2015- the effect of maternal human immunodeficiency virus status.

[17] Saffron, Lale Say, João-Paulo Souza, Carol J Hogue, Dinorah L Calles, A Metin Gülmezoglu and Rosalind Raine: The relationship between maternal education and mortality among women giving birth in health care institutions: Analysis of the cross sectional WHO Global Survey on Maternal and Perinatal Health; BMC Public Health2011 11:606, doi:10.1186/1471-2458-11-60 10.1186/1471-2458-11-606 PMC3162526 21801399

[18] Abou-Ali H (2014). The effect of water and sanitation on child mortality in Egypt environmental economics unit.

[19] Mostafa A. Arafa, Taher Amine and Moataz Abdel (2007): Fattah Association of Maternal Work with Adverse Perinatal Outcome *Canadian Journal of Public Health / Revue Canadienne de Santé Publique* Vol. 98, No. 3 , pp. 217–221. 10.1007/BF03403716 PMC6975771 17626388

